# Electronic Health Records, Health Outcomes, and Vital Statistics: Opportunities and Challenges

**DOI:** 10.1016/j.mayocpiqo.2024.08.005

**Published:** 2024-10-08

**Authors:** Aaron C. Spaulding

**Affiliations:** From the Division of Health Care Delivery Research, Robert D. and Patricia E. Kern Center for the Science of Health Care Delivery, Mayo Clinic, Jacksonville, FL

Evaluation of health service intervention is crucial for determining the best care paths for patients and health care organizations. In health service research studies, validity is a vital component defining how well the study results represent the “truth.” When our studies cannot correctly measure constructs, evaluate causal relationships, adhere to statistical principles, or demonstrate relevance to entities outside the study, the value of the work and our faith in the conclusions are limited. As a research community, it is in our best interest to evaluate interventions to improve care as well as the tools and measures by which we assess our interventions.

The work by Yokoi et al^1^ provides such a look. Their study evaluated the accuracy of mortality data in 500 patients who underwent coronary artery bypass grafting. Results demonstrate discordance between the electronic medical record (EMR) and direct outreach collection, with the EMR overestimating the mortality rate. Clearly, this reduces the validity of studies relying on data points necessary to determine the safety and long-term outcomes of the care or intervention provided.

Although Yokoi et al^1^ provide a clear and current example of the difference in data points, they are not the first to describe differences between the EMR and other data collection methods. For example, previous work includes an assessment of the agreement between registry and administrative data sets in defining comorbidities,^2^ as well as the rates of postoperative complications between administrative and clinical data sources.^3^ In each case, inconsistencies are identified on the basis of the nuances and purposes for which these data sets are created and used. The root of the problem, at least as far as using it for secondary purposes, is often on the basis of the original intent or reason for the use of the data set. Clearly, the EMR was not initially designed to be a death registry, although mortality end points are important and likely to be accurate in the short term. To that end, we must consider the strengths and weaknesses of the data sources as they influence our ability to make solid inferences for research.

It is also important to note that, in large part, we have made tremendous strides in our abilities to collect data from multiple sources that ultimately allow us to improve understanding and gain a fuller picture. In the United States, there is a continued focus on interoperability and the need for our various health technologies to be able to “speak” to each other. Implementing the Health Information Technology for Economic and Clinical Health (HITECH) act in 2009 focused on accelerating electronic health record adoption and meaningful use criteria directly apply to ensuring our data points are available, robust, and usable.^4^ Improvements in the availability of health information technology have again improved our ability to acquire data from multiple sources; however, although interoperability has improved, there is still much work needed because we are yet to develop consistent and broad-reaching health information exchanges that would address problems such as those described by Yokoi et al.^5^^,^^6^

Nevertheless, improvements in data access and use cannot be the end point. We must ensure that the data points we rely on are valid. For example, before 2011, the United States had a more robust Social Security Death Index created from the US Social Security Administration’s Master Death File, which could be and often was used to address the specific issues posed by Yokoi et al.^7^ In short, using the Social Security Administration files, which each US State populated, researchers could ascertain the status of patients in their studies given the appropriate administrative approvals. This allowed for evaluating mortality outcomes that are otherwise potentially unknown given the more acute interactions, which generally populate the data in our EMRs. However, on the basis of changes occurring from 2011, the information provided by individual state sources is no longer included. As a result, multiple studies have now identified that the Death Files are no longer valid for research purposes.^8^^,^^9^ Although administrative costs and privacy protections were of concern, the loss of such a resource negatively influences the ability to gain valid outcome information and pair it with the robust data generally collected during an acute health care interaction.

Finally, and in closing, we gather more information today than ever before, and there are many attempts to leverage that data to improve care and efficiency within our health systems. With the continued and escalating use of artificial intelligence, machine learning, and large data models, we must consider the validity and robustness of the data we use for any research or data modeling. Developing avenues to achieve the goals described by Yokoi et al to develop an enhanced follow-up strategy or to build valid data repositories that document issues, such as mortality, can only improve the ability of our studies to make appropriate inferences and to ensure that the interventions being studied are on the basis of a foundation of valid evidence. We all do better when we better understand the influence of interventions on our health. It is difficult to do that when systems designed to benefit the population cannot be queried or linked.

## Potential Competing Interest

Given his role as Editorial Board Member, Dr Spaulding had no involvement in the peer-review of this article and has no access to information regarding its peer-review. The author has no disclosures to report.

## References

[1] Yokoi K., Tanaka A., Yoshioka G. (2024). Accuracy of outcome ascertainment in long-term mortality after coronary bypass grafting. Mayo Clin Proc Innov Qual Outcomes.

[2] Etzioni D.A., Lessow C., Bordeianou L.G. (2020). Concordance between registry and administrative data in the determination of comorbidity: a multi-institutional study. Ann Surg.

[3] Koch C.G., Li L., Hixson E., Phillips S., Henderson J.M. (2012). What are the real rates of postoperative complications: elucidating inconsistencies between administrative and clinical data sources. J Am Coll Surg.

[4] Adler-Milstein J., Jha A.K. (2017). HITECH act drove large gains in hospital electronic health record adoption. Health Aff (Millwood).

[5] Bates D.W., Samal L. (2018). Interoperability: what is it, how can we make it work for clinicians, and how should we measure it in the future?. Health Serv Res.

[6] Kadakia K.T., Howell M.D., DeSalvo K.B. (2021). Modernizing public health data systems: lessons from the Health Information Technology for Economic and Clinical Health (HITECH) act. JAMA.

[7] da Graca B., Filardo G., Nicewander D. (2013). Consequences for healthcare quality and research of the exclusion of records from the Death Master File. Circ Cardiovasc Qual Outcomes.

[8] Levin M.A., Lin H.-M., Prabhakar G., McCormick P.J., Egorova N.N. (2019). Alive or dead: validity of the Social Security Administration Death Master File after 2011. Health Serv Res.

[9] Navar A.M., Peterson E.D., Steen D.L. (2019). Evaluation of mortality data from the social security administration death master file for clinical research. JAMA Cardiol.

